# The ability to interpret courtship signals in patients with schizophrenia

**DOI:** 10.1192/j.eurpsy.2025.2215

**Published:** 2025-08-26

**Authors:** P. Biedkova, O. Vaníček, K. Ständer, E. Kolářová, R. Androvičová

**Affiliations:** 1 Charles University; 2 Centre for sexual health and interventions, National Institute of Mental Health, Prague, Czech Republic; 3 Amsterdam Medical Centre, Amsterdam, Netherlands

## Abstract

**Introduction:**

Numerous studies have highlighted the unmet needs in intimate and sexual relationships among patients with schizophrenia. Although research shows that patients with schizophrenia express a desire for romantic and sexual connections, they encounter difficulties in forming and sustaining these relationships. However, the underlying causes of deficits in sexual and romantic functioning remain unknown and unexplored.

**Objectives:**

The study aims to assess whether difficulties in forming intimate relationships could be connected to a decreased ability to interpret courtship signals.

**Methods:**

49 male participants (36 patients, 13 controls) and 27 female participants (14 patients, 13 controls) were exposed to our experiment „Does she like me?” task. The task involves a video stimuli that depicts a woman/man displaying different nonverbal behaviors (rejection, courtship, and neutral attitude). Each participant evaluated the woman’s sexual interest/ man’s sexual interest on a nine-point scale. In addition, eye movements and pupil dilation were measured using the eye-tracking device Eyelink 1000plus. Patients and controls were compared on subjective report and psychophysiological eye tracking markers. For data analysis, we used Repeated measures ANOVA.

**Results:**

There was no statistically significant difference between the control group and patients in their assessment of women´s interest (F (2, 94) = 1.28, p = 0.28, η_p_^2^= 0.03) or men’s interest (F (2,50) = 0.24, p = 0.79, η_p_^2^= 0.01). Also, there was no difference in average pupil size. However, while both groups correctly identified the nonverbal cues and responded accordingly (with the greatest assessments of interest for positive stimuli, lesser for neutral stimuli, and the least for negative stimuli), male patients on average tended to overestimate women’s interest in all conditions (positive, neutral, negative) compared to the control group.

**Conclusions:**

According to our results, the ability to interpret courtship signals does not differ significantly between patients and controls. However, male patients tend to overestimate women’s sexual interest on average, which may be one of the reasons why they face challenges in intimate relationships. Further research is needed to explore this.

The study was supported by the Czech Health Research Council, no. NU21J-04-00024 and by the Charles University, Fac Med1, GAUK no. 56123.

**Disclosure of Interest:**

None Declared

